# Adrenal volumes in fetuses delivering prior to 32 weeks' gestation: An MRI pilot study

**DOI:** 10.1111/aogs.14733

**Published:** 2023-11-27

**Authors:** Megan Hall, Jana Hutter, Alena Uus, Elise du Crest, Alexia Egloff, Natalie Suff, Mudher Al Adnani, Paul T. Seed, Deena Gibbons, Maria Deprez, Rachel M. Tribe, Andrew Shennan, Mary Rutherford, Lisa Story

**Affiliations:** ^1^ Center for the Developing Brain St Thomas' Hospital, King's College London London UK; ^2^ Department of Women and Children's Health St Thomas' Hospital, King's College London London UK; ^3^ Department of Cellular Pathology St Thomas' Hospital, Guy's and St Thomas' NHS Foundation Trust London UK; ^4^ Department of Immunobiology King's College London London UK

**Keywords:** adrenal gland, chorioamnionitis, fetal inflammatory response syndrome, fetal MRI, preterm birth, preterm prelabor rupture of the membranes

## Abstract

**Introduction:**

Spontaneous preterm birth prior to 32 weeks' gestation accounts for 1% of all deliveries and is associated with high rates of morbidity and mortality. A total of 70% are associated with chorioamnionitis which increases the incidence of morbidity, but for which there is no noninvasive antenatal test. Fetal adrenal glands produce cortisol and dehydroepiandosterone‐sulphate which upregulate prior to spontaneous preterm birth. Ultrasound suggests that adrenal volumes may increase prior to preterm birth, but studies are limited. This study aimed to: (i) demonstrate reproducibility of magnetic resonance imaging (MRI) derived adrenal volumetry; (ii) derive normal ranges of total adrenal volumes, and adrenal: body volume for normal; (iii) compare with those who have spontaneous very preterm birth; and (iv) correlate with histopathological chorioamnionitis.

**Material and methods:**

Patients at high risk of preterm birth prior to 32 weeks were prospectively recruited, and included if they did deliver prior to 32 weeks; a control group who delivered an uncomplicated pregnancy at term was also recruited. T2 weighted images of the entire uterus were obtained, and a deformable slice‐to‐volume method was used to reconstruct the fetal abdomen. Adrenal and body volumes were obtained via manual segmentation, and adrenal: body volume ratios generated. Normal ranges were created using control data. Differences between groups were investigated accounting for the effect of gestation by use of regression analysis. Placental histopathology was reviewed for pregnancies delivering preterm.

**Results:**

A total of 56 controls and 26 cases were included in the analysis. Volumetry was consistent between observers. Adrenal volumes were not higher in the case group (*p* = 0.2); adrenal: body volume ratios were higher (*p* = 0.011), persisting in the presence of chorioamnionitis (*p* = 0.017). A cluster of three pairs of adrenal glands below the fifth centile were noted among the cases all of whom had a protracted period at risk of preterm birth prior to MRI.

**Conclusions:**

Adrenal: body volume ratios are significantly larger in fetuses who go on to deliver preterm than those delivering at term. Adrenal volumes were not significantly larger, we hypothesize that this could be due to an adrenal atrophy in fetuses with fulminating chorioamnionitis. A straightforward relationship of adrenal size being increased prior to preterm birth should not be assumed.

## Graphical abstract

Volumetric assessment of the fetal adrenal using MRI is technically achievable In fetuses who go on to deliver extremely preterm, an increased adrenal:body volume is seen.

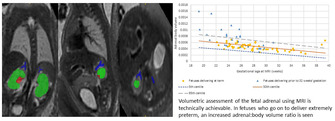

**Abbreviations**
MRI: magnetic resonance imaging

**Key message**

Adrenal volumes can be reliably calculated on MRI. An increase in adrenal: body volume ratio occurs in fetuses who go on to deliver preterm, adrenal volumes are not consistently increased perhaps because of an atrophic process associated with preterm birth pathology.

## 1 INTRODUCTION

Very preterm birth before 32 weeks of gestation accounts for 1.2% of all deliveries in the UK^1^ and is associated with significant short‐ and long‐term morbidity, as well as perinatal mortality.^2^ Between 40% and 70% of spontaneous preterm births are associated with chorioamnionitis,^3^ although this is inversely associated with gestation with the incidence rising to 94% when delivery occurs prior to 24 weeks' gestation.^4^ Chorioamnionitis is a histopathological diagnosis that is divided into a maternal immunological and a fetal inflammatory response.^5^ Chorioamnionitis is associated with poorer neurological and respiratory outcomes, and a higher rate of necrotizing enterocolitis than in gestation matched controls.^6^

There is currently no diagnostic noninvasive antenatal test for chorioamnionitis, and routine clinical practice involves monitoring for maternal signs and symptoms, which correlate poorly with histopathological diagnosis or outcome.^3^ Components of the fetal immune system, such as the thymus, have been investigated as potential markers of fetal infection with promising results,^7^ although this is yet to be translated into clincal practice. The adrenal gland, which sits above the kidney, has a significant role in steroidogenesis and plays a role in stress response to inflammation by production of cortisol. Given the inflammatory processes the fetus undergoes secondary to chorioamnionitis, assessment of the fetal adrenal glands may prove a valuable diagnostic tool.

While ultrasound assessment of the adrenals has been investigated as a biomarker of both placental insufficiency^8^ and preterm birth,^9^, ^10^ this has never been integrated into clinical practice, perhaps because of difficulty replicating results in a small and irregularly shaped organ and heterogeneity of populations investigated. Normal length and thickness of adrenal glands in healthy fetuses has been derived from fetal magnetic resonance imaging (MRI),^11^ but volumetric ranges have been suggested based on post‐mortem cases only.^12^

We hypothesize that increased adrenal activation prior to spontaneous preterm will result in gland enlargement. To test this, the aims of this study were to (i) demonstrate reproducibility of MRI derived adrenal volumetry; (ii) derive normal ranges of total adrenal volume, and adrenal to body volume for fetuses with normal outcomes; (iii) compare these to those who have had spontaneous very preterm birth; and (iv) correlate with those who have a histopathological diagnosis of chorioamnionitis with or without funisitis.

## 2 MATERIAL AND METHODS

Pregnant patients between 18 and 31^+6^ weeks’ gestation, considered to be at high risk of preterm birth, were prospectively recruited from a large tertiary London hospital between December 2015 and April 2022 as part of an observational study. Inclusion criteria were preterm prelabor rupture of the membranes (PPROM), exposed membranes, or a > 75% risk of delivery before 32 weeks' gestation based on cervical length, quantitative fetal fibronectin and pre‐existing risk factors for spontaneous preterm birth based on the QUIPP app score.^13^ Exclusion criteria were contraindication to MRI, any other obstetric complications such as hypertension or diabetes, twin pregnancy, chromosomal or structural abnormalities, active labor, and inability to given informed consent.

The control group was retrospectively obtained from low‐risk pregnancies that delivered at term where woman had volunteered to have an MRI for research as controls. All cases followed the protocols and post processing techniques described below.

Fetal MRI was obtained on a Phillips Achieva 3T system with a 32‐channel coil. Imaging was obtained with patients in a supported supine position and with continuous heart rate and oxygen saturation monitoring. Total scan length did not exceed 1 h, with a short break offered halfway through the examination if needed.

Imaging of the fetal body was acquired using 2D T2 weighted single‐shot turbo spin echo in between three and seven orthogonal planes reorientated with respect to the maternal habitus or the fetal body and brain. The following scanning parameters were used: TR = 25 991 ms, TE 180 ms; slice thickness 2.4 mm; slice overlap 1.25 mm and in‐plane resolution of 1.251 × 1.251 × 1.21 mm; flip angle = 90^0^. Three‐dimensional MR images with 0.8 mm isotropic resolution of the fetal abdomen were obtained from the resulting motion corrupted T2‐weighted MRI stacks using deformable slice‐to‐volume reconstruction to correct for motion artifacts.^14^

**FIGURE 1:**
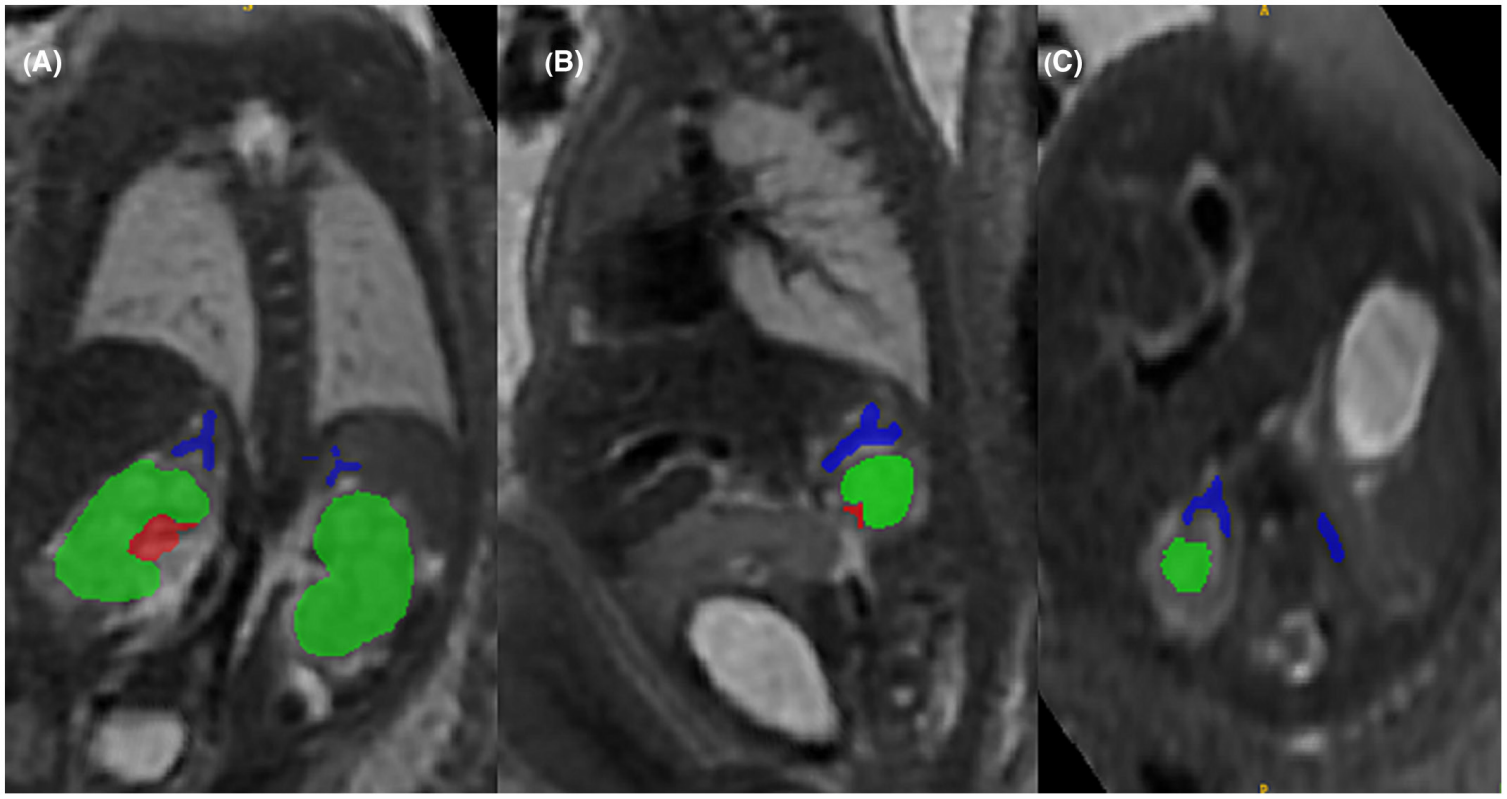
Adrenal segmentation in a control fetus at 31^+6^ weeks' gestation. Adrenal gland: blue; renal parenchyma: green; renal collecting system: red. (A) coronal, (B) sagittal, (C) axial.

The fetal adrenals were segmented manually and reconstructed using ITK‐SNAP version 3.6.0 (Figure 1).^15^ Intra‐ and interobserver variability was confirmed by two experienced operators (authors MH, 3 years' fetal MRI experience, and LS, 12 years' fetal MRI experience). Difficult cases were reviewed by a specialist perinatal radiologist with expertise in fetal MRI (author AE, 16 years' experience). Body volume was also calculated via manual segmentation and reconstruction on ITK‐SNAP.

Collected outcome data included maternal demographics, maternal steroid administration, gestation at delivery, sex of infant, birthweight, birthweight centile (via INTERGROWTH‐21 charts), neonatal unit admission, number of days of intensive and high dependency care, invasive ventilation, continuous positive airway pressure, supplemental oxygen, sepsis and necrotizing enterocolitis. A composite outcome score consisting of intraventricular hemorrhage grade 3–4, persistent periventricular echo densities, necrotizing enterocolitis, chronic lung disease (defined as supplementary oxygen requirement at 36 weeks' corrected gestation), and retinopathy of prematurity requiring treatment was calculated for all neonates who survived to discharge.^16^ Where available, placental histopathology for a patient who delivered preterm was reviewed by a specialist perinatal histopathologist, with chorioamnionitis and funisitis defined as per the Amsterdam criteria.^5^

### 2.1 Statistical analyses

Two‐way random effect intraclass correlation coefficients were used to confirm intra‐ and interobserver consistency. Normality was assessed using the Kolmogorov–Smirnov method. For the control group, the Royston and Wright method^17^ was used to estimate normal growth trajectory of adrenal gland volume and adrenal: body ratios between 20 and 35 weeks' gestation. Demographic and neonatal outcome data were analyzed using student *t* test where data were continuous, and chi‐squared test were categorical. Differences between groups were investigated accounting for the effect of gestation by use of regression analysis. Statistical analysis was undertaken in SPSS version 28; centile graphs were then created using Microsoft Excel.

## 3 RESULTS

**FIGURE 2:**
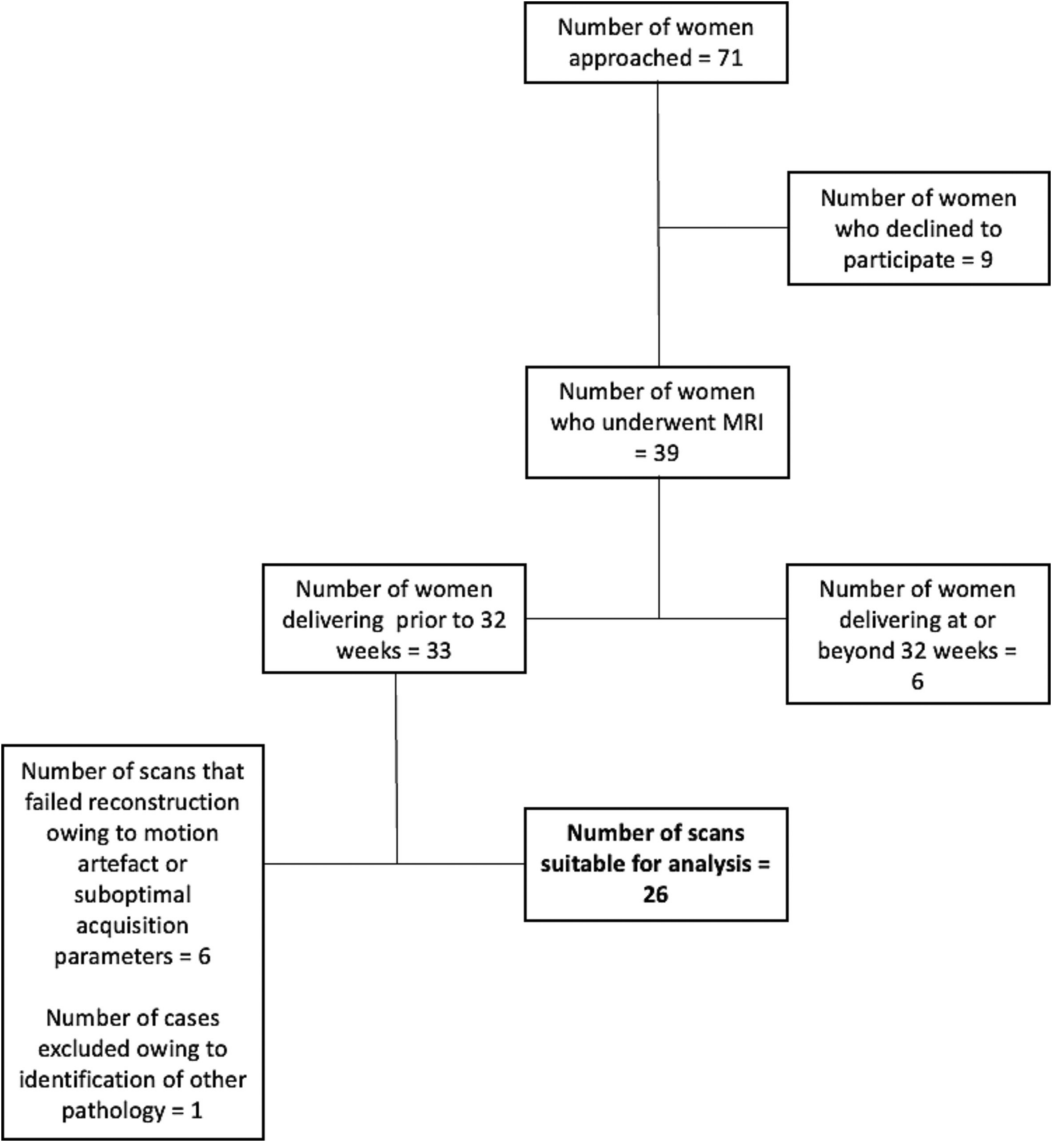
Flow chart illustrating high risk recruitment.

Recruitment of high‐risk cases is summarized in Figure 2. Of the 26 patients analyzed, 14 had PPROM prior to MRI. Prior to MRI, 14 patients had received a complete course of antenatal corticosteroids, five patients had a single dose, and seven patients had not received any doses. Placental histopathology was available for 23 cases: 20 demonstrated chorioamnionitis, with 14 also having funisitis. The mean time from MRI to delivery was 9.65 days (SD 9.47).

A total of 56 control cases were identified, all of whom delivered after 37 weeks of gestation without any maternal or fetal complications. Clinical characteristics of cases and controls are given in Table 1.

**TABLE 1:** Clinical characteristics of cohort.

Characteristic	Term cohort (*n* = 56)	Preterm cohort (*n* = 26)	*p*‐value (95% CI)
Maternal age (year) Mean (SD)	33.57 (4.08)	32.57 (5.33)	0.21 (−0.261 to 0.674)
BMI (kg/m^2^) Mean (SD)	22.42 (2.28)	23.87 (3.50)	0.22 (−2.73 to 0.38)
Ethnicity, *n* (%)			
White	44 (79)	15 (58)	
Black	6 (11)	4 (15)	
South Asian	2 (4)	5 (19)	
East Asian	1 (2)	1 (4)	
Other	2 (4)	1 (4)	
Missing	1 (2)	0	
Ethnicity grouping, *n* (%)			
White	44 (79)	15 (58)	0.04
Non‐white	11 (20)	11 (42)	
Parity, *n* (%)			
Primiparous	38 (68)	14 (54)	0.22
Multiparous	18 (32)	12 (46)	
GA at MRI (week) Mean (SD)	28.31 (4.18)	25.21 (3.32)	0.002 (1.13 to 4.8)
GA at birth (week) Mean (SD)	39.40 (1.29)	26.47 (3.42)	
Birthweight (g) Mean (SD)	3433 (426.70)	993.38 (390.09)	
Range (g)	2485 to 4400	360 to 1865	
Birthweight centiles of live births, median (interquartile range)^a^	63 (35 to 83)	57 (53 to 81)	
Sex of infant, *n* (%)			
Female	23 (50)	15 (58)	0.61
Male	23 (50)	11 (42)	
Undetermined	0	0	
Outcome, *n* (%)			
Live at discharge	60 (100)	18 (69)	<0.001
Death prior to discharge	0	8 (31)	
Second trimester pregnancy loss	0	5 (19)	
Neonatal death	0	3 (12)	

*Note*: Continuous data analyzed using student's *t* test; categorical data using Chi^2^.

Abbreviations: BMI, body mass index; CI, confidence interval; GA, gestational age; MRI, magnetic resonance imaging.

^a^ Centiles not available for deliveries <23 weeks' gestation.

**FIGURE 3:**
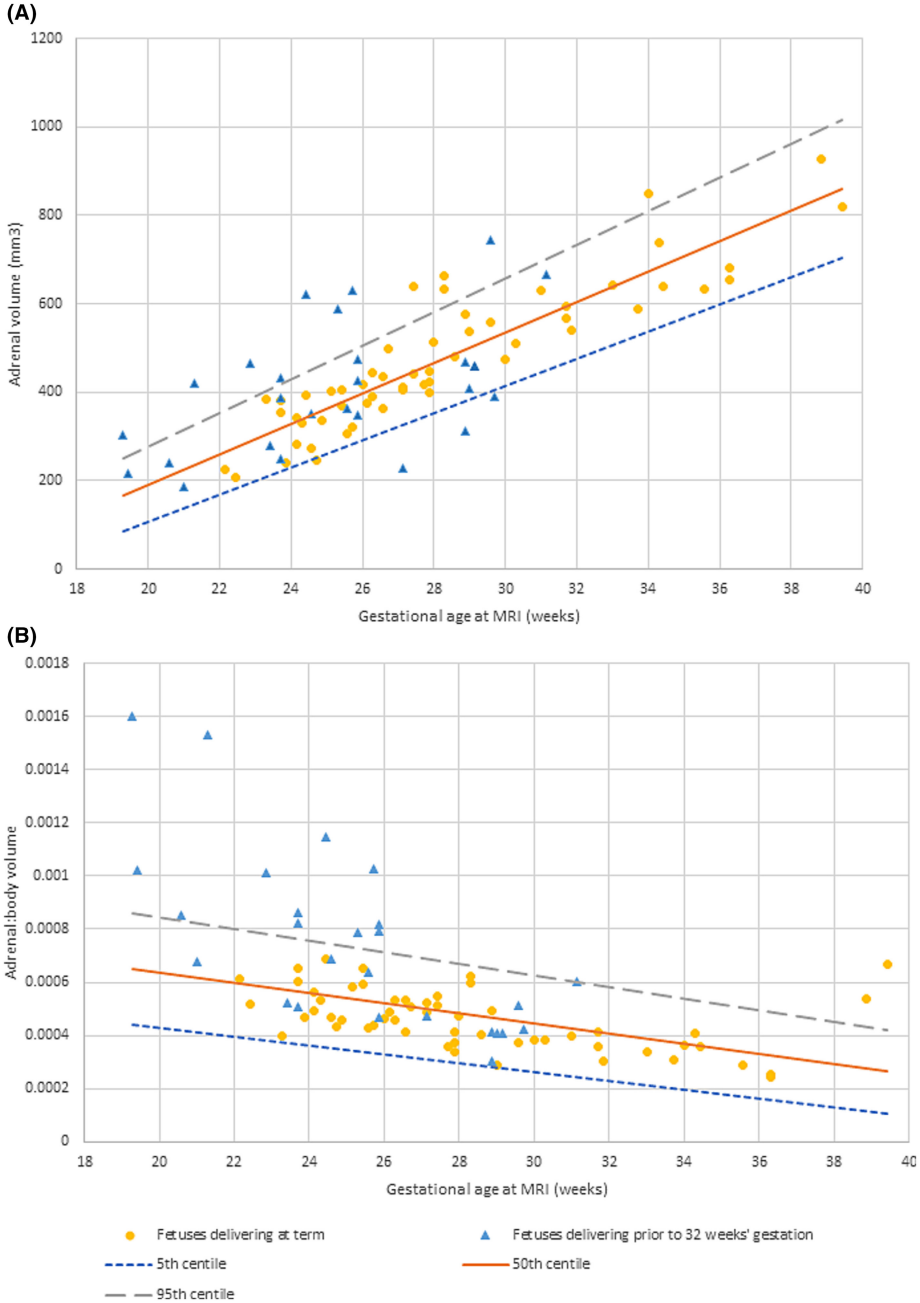
Magnetic resonance imaging (MRI) derived normal ranges (with 50th centile, 5th centile and 95th centile lines as indicated) of (A) fetal adrenal volumes and (B) fetal adrenal: body volume ratios in fetuses who subsequently deliver at term and those who deliver preterm. Centiles were calculated using the Royston and Wright method.

**FIGURE 4:**
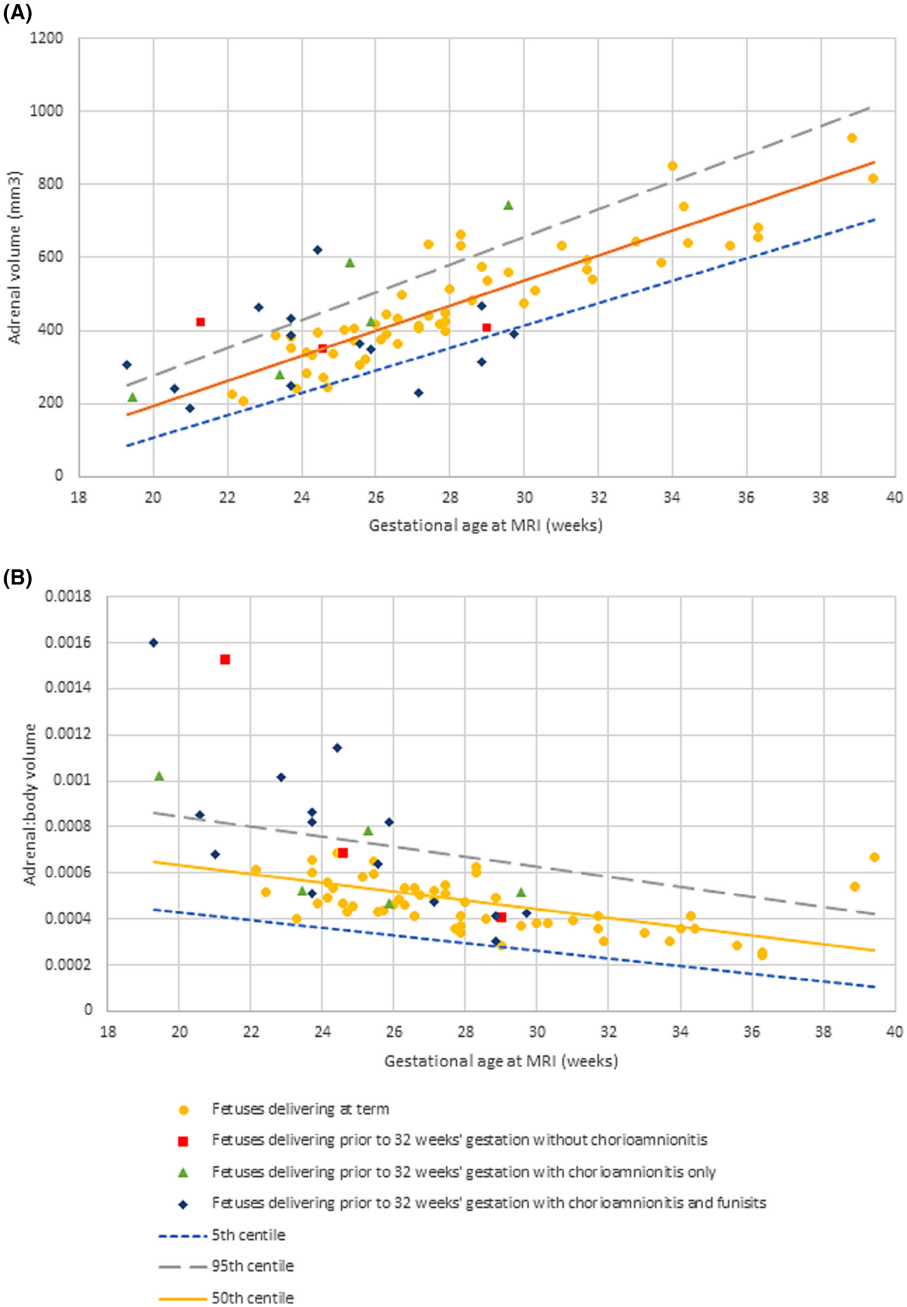
Magnetic resonance imaging (MRI) derived normal ranges (with 50th centile, 5th centile and 95th centile lines as indicated) of (A) fetal adrenal volumes and (B) fetal adrenal: body volume ratios in fetuses who subsequently deliver preterm (<32 weeks' gestation) with and without chorioamnionitis and funisits as compared to term.

Segmentation was undertaken with acceptable intra‐ and interobserver variability (intraobserver variability = 0.96; interobserver variability = 0.97). The distribution of total adrenal volumes and adrenal: body ratios for all controls can be seen in Figure 3 alongside derived centile lines. Cases that delivered preterm had significantly higher adrenal: body volume ratios (*p* = 0.011; 95% CI: 0–0.001), although this did not persist for adrenal volumes alone (*p* = 0.2) (Figure 3). Similarly, adrenal: body volumes were significantly higher in cases that went on to be diagnosed with chorioamnionitis (with or without funisitis), although numbers are small (*p* = 0.017; 95% CI: 0–0.001) (Figure 4). Consistent with previous work, body volumes of fetuses who were born preterm were smaller than the control group (*p* < 0.001).^18^, ^19^ Given the impact of exogenous steroids on adrenal function, this relationship was explored, but no effect was detected (adrenal volumes *p* = 0.558; adrenal: body volumes *p* = 0.889).

Possible causes of adrenal gland volumes <5th centile were considered as these cases were unexpected in this patient group although there was no difference in timing of antenatal corticosteroids or composite neonatal outcomes at discharge for this group. All three were diagnosed with chorioamnionitis and funisitis, although this was not unique to this group. It was noted that all three cases had a prolonged antenatal course deemed to be at high risk of preterm delivery prior to MRI, and delivered either spontaneously or iatrogenically for suspected chorioamnionitis very shortly after the MRI.

There were 5 s trimester pregnancy losses and three neonatal deaths among the high‐risk group secondary to complications of severe prematurity. Of the infants who survived to discharge, two had composite neonatal outcome scores of two; six of one; and all remaining infants scored 0. There were no adverse neonatal outcomes or admissions to the neonatal unit among the control group.

## 4 DISCUSSION

In this study, we demonstrate feasibility of visualization of fetal adrenal glands on MRI, with assessment of volume that is clinically acceptable in its reproducibility^20^; report normal range of total fetal adrenal volumes and show a positive relationship between adrenal gland volume and gestational age, alongside a decrease in adrenal to body volume. Adrenal volume: body ratio seems to increase around term, likely due to physiological changes around labor, although a larger sample would be required to confirm this. Adrenal to body volume was higher in pregnancies that went on to deliver preterm, although the same did not hold true of adrenal volumes alone.

A study of 204 fetuses generated normal ranges for the fetal adrenal gland using virtual organ computer aided analysis (VOCAL, a 3D ultrasound technique) for healthy fetuses from 24 to 37^+6^ weeks' gestation.^21^ Similar to our study, they demonstrate forward organ growth with gestation; the same relationship holds true when 3D gland volume is predicted using a manual polynomial formula rather than VOCAL.^22^ While this could be a useful technique, image acquisition is restricted by oligohydramnios, making it less clinically relevant to patients at high risk of preterm labor. Nonetheless, ultrasound assessment of the fetal adrenal for the prediction of preterm birth in high risk patients has been attempted. While three studies have previously demonstrated either whole gland or fetal zone enlargement, the use of clinically unavailable techniques, or need for highly experienced sonographers, are likely to have hindered clinical translatability.^10^, ^23^, ^24^

To our knowledge, two previous studies have attempted to assess the normal fetal adrenal gland on MRI: one used single coronal images to determine a length and thickness to the gland^11^; however, there was no consistency in slice used and it is unclear which measurements of the adrenal are most affected by volumetric change rendering this less relevant. Assessment of whole volumes is likely be more applicable to clincial practice. A second study assessed postmortem fetal adrenal volumes,^12^ although the value of postmortem volumes is limited by changes in gland function and size during pathological processes prior to death.

One human study has demonstrated that fetuses born with intramniotic inflammation have higher adrenal volumes as well as higher amniotic fluid dehydroepiandrosterone‐sulfate (DHEAS) and cortisol, suggesting hypothalamic–pituitary–adrenal activation as a mechanisms of gland enlargement^25^; a finding further supported by evidence of increased DHEAS and cortisol in patients who deliver preterm with intramniotic infection than without.^26^ These findings corroborate our work by suggesting upregulation of adrenal function in fetuses at high risk of preterm birth.

**FIGURE 5:**
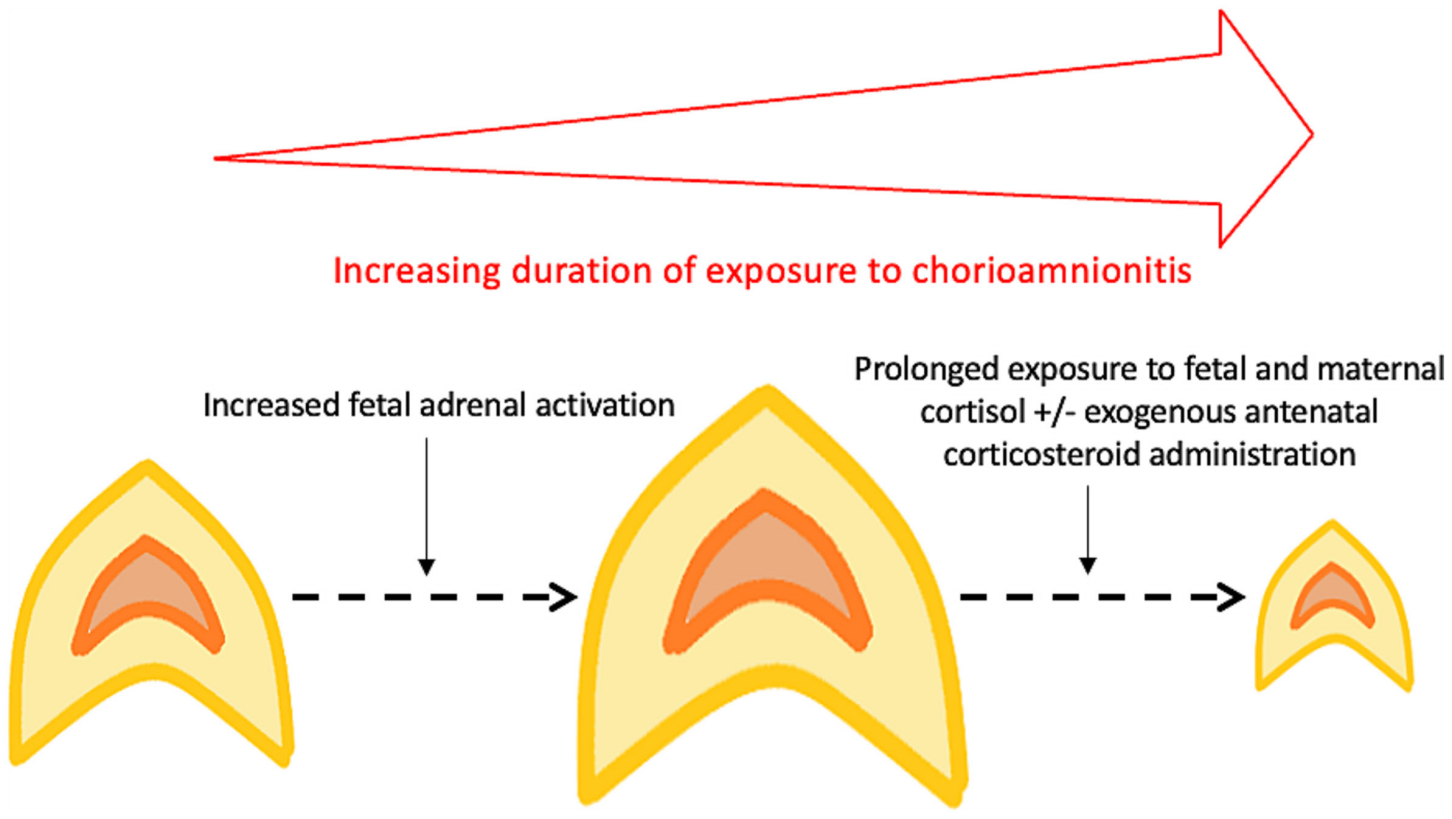
Hypothesized mechanism of reduction in adrenal size secondary to exposure to chorioamnionitis.

An unexpected finding in our study was a cluster of adrenal gland volumes in three fetuses who delivered preterm below the fifth centile. We propose that this is secondary to fulminating chorioamnionitis: all cases had protracted periods where they were deemed to be at high risk of preterm labor and delivered shortly after MRI; all three were found to have chorioamnoinitis and funisitis. This is supported by neonatal findings demonstrating smaller adrenal glands in a chorioamnionitis exposed group compared to gestation matched unaffected neonates.^27^ No study has analyzed postmortem adrenal weights of infants exposed to chorioamnoinitits, although there is evidence of cortical lipid depletion and cytolytic degeneration.^28^ In adults exposed to sepsis prior to death, adrenal atrophy is an expected postmortem finding in cases with exposure to steroids.^29^ As well as exposure to endogenous steroids that are not subject to normal negative feedback loops, fetuses with protracted chorioamnionitis may also be exposed to increased maternal steroids (related to both an immune response to inflammation^30^ and placental overproduction of corticotropin‐releasing hormone [CRH]) and exogenous antenatal corticosteroids: it is conceivable that a similar mechanism is at work in this group (Figure 5).

This is the first MRI study to give volumetric normal ranges of the fetal adrenal glands, and the first to compare to cases of spontaneous preterm delivery. MRI is likely to be valuable over ultrasound in this case given the relative size of the adrenals at earlier gestations, and the difficulty in obtaining high quality anatomical data on ultrasound in any case of oligohydramnios. The application of deformable slice‐to‐volume reconstruction increases the reliability, as does review of the images by a senior clinician very experienced in fetal body MRI.

However, the sample size is small for both cases and controls, and the validity of results would be improved with a larger dataset including a particular focus at earlier gestational ages among the controls. All scans were undertaken in a single unit, and not all cases had placental histopathology. Owing to national protocols on antenatal corticosteroid administration, it was not possible to meaningfully control for this variable. Alongside antenatal corticosteroids, a multiple logistic regression could be performed using a larger sample size and considering data such as cervical length and inflammatory markers. Many datasets were obtained in studies where body imaging was not the primary purpose, and so only 74% of images reviewed were suitable for analysis; however, this has increased to 89% more recently, a number we anticipate will continue to rise. There is a slight disparity in ethnicities between the cases and controls which must be addressed in order to make these findings more universally applicable.

While we agree that enlarged fetal adrenals appear to occur in fetuses who go on to deliver preterm, further work is required to establish if small adrenals (<5th centile) is indicative of a more severe phenotype which could be useful in determining timing of delivery. Whether these cases had a period of enlargement is unclear and acquisition of longitudinal data would help explore this. Within an increased sample, further work should be done correlating results to placental histopathology, as well as short‐ and long‐term neonatal outcomes. There is currently no evidence regarding the impact of corticosteroid administration on fetuses who subsequently do not deliver preterm, and this would be of interest in better understanding the impact of iatrogenic steroids alone. There could be a role for correlation with amniotic fluid adrenal hormones, but this avenue is confounded by placental derived adrenal hormones. As well as further optimizing image techniques and developing automatic segmentation pipelines, application of functional imaging techniques may reveal further changes associated with preterm birth and chorioamnionitis; this alongside volume can be considered as part of a larger model for prediction of preterm chorioamnionitis. Assessment in conjunction with the fetal thymus, which is a major immune organ in the fetus, may give further information on the interaction between immune and stress responses.

## 5 CONCLUSION

Assessment of fetal adrenal gland volume is achievable in a clinically reliable manner. Adrenal gland to body volume ratio is increased in fetuses who go on to deliver extremely preterm. This work supports adrenal assessment as part of a tool for prediction of chorioamnionitis and preterm birth; but application of adrenal volumes alone into clinical practice, as has been previously suggested, should not be undertaken until further investigation of the potential finding of atrophy has been further explored.

## AUTHOR CONTRIBUTIONS

MH: conceptualization, data curation, formal analysis, methodology, investigation, writing—original draft; JH: data curation, formal analysis, methodology, investigation, funding acquisition, writing—review and editing; AU: formal analysis, methodology, investigation, writing—review and editing; EC: data curation, writing—review and editing; AE: data curation, writing —review and editing; NS: supervision, writing—reading and editing; MAA: investigation, writing—review and editing; PTS: formal analysis, writing—review and editing; DG: supervision, writing—reading and editing; MD: investigation, writing—review and editing; RMT: supervision, writing—reading and editing; AS: supervision, writing—review and editing; MR: supervision, writing—reading and editing; LS: conceptualisation, data curation, formal analysis, funding acquisition, investigation, methodology, supervision, writing—review and editing.

## FUNDING INFORMATION

This work was supported by core funding from the Wellcome/EPSRC Center for Medical Engineering (WT203148/Z/16/Z), by the NIH Human Placenta Project grant 1U01HD087202–01 (Placenta Imaging Project [PIP]), by the Wellcome Trust, Sir Henry Wellcome Fellowship to JH, [201 374/Z/16/Z], by the UKRI, FLF to JH [MR/T018119/1] and by the National Institute for Health Research (NIHR) Biomedical Research Center based at Guy's and St Thomas' NHS Foundation Trust and King's College London. LS is funded by the National Institute for Health Research (NIHR) (NIHR Advanced Fellowship [301664]). The views expressed are those of the authors and not necessarily those of the NHS, the NIHR or the Department of Health.

## CONFLICT OF INTEREST STATEMENT

The authors report no conflict of interest.

## Ethics statement

Ethical approval was obtained under 16/LO/1573 (September 23, 2016, the placental imaging project), 19/SS/0032 (March 7, 2019, antenatal assessment of fetal infection using advanced MRI protocols) and 21/SS/0082 (November 29, 2021, individual risk prediction of adverse neonatal outcomes in pregnancies that deliver preterm using advanced MRI techniques and machine learning). All regulatory approvals were obtained prior to commencing research. Written consent was obtained prior to commencing scanning or data collection.
